# The Neurosurgical Intraoperative Checklist for Surgery of the Craniocervical Junction and Spine

**DOI:** 10.7759/cureus.7588

**Published:** 2020-04-08

**Authors:** Fraser Henderson, Robert Rosenbaum, Malini Narayanan, John Mackall, Clayton Korson

**Affiliations:** 1 Neurological Surgery, University of Maryland Prince George's Hospital Center, Largo, USA; 2 Neurological Surgery, Doctors Community Hospital, Lanham, USA; 3 Neurological Surgery, The Metropolitan Neurosurgery Group, Silver Spring, USA; 4 Neurological Surgery, Walter Reed National Military Medical Center, Bethesda, USA; 5 Neurological Surgery, University of Maryland Prince George’s Hospital Center, Cheverley, USA; 6 Neurological Surgery, D&K Medical, LLC., Lanham, USA; 7 Emergency Medicine, Creighton University School of Medicine, Omaha, USA

**Keywords:** checklist, neurosurgery, spine, craniocervical junction, quality control, iatrogenic complication

## Abstract

Many sectors within healthcare have adapted checklists to improve quality control. Notwithstanding the reported successful implementation of surgical checklists in the operating theater, a dearth of literature addresses the specific challenges posed by complex surgery in the craniocervical junction and spine. The authors devised an intraoperative checklist to address the common errors and verify the completion of objectives unique to these surgeries. The data over six years is presented retrospectively; no historical control for comparison is available, as those omissions and surgical errors addressed by the checklist are not generally registered in any morbidity and mortality reports. Through six years and approximately 1200 surgeries, the checklist was implemented with 98% compliance. The checklist eliminated the occurrences of mundane surgical errors, minimized iatrogenic complications, and ensured completion of specific objectives. We discuss that preoperative checklists, now in general use in all hospitals, have not addressed the most common, intraoperative omissions. These technical omissions result in part from the complexity of spine surgery and directly impact the surgical outcome. The Neurosurgical Intraoperative Checklist is a practical, rapid, and comprehensive means to prevent common, avoidable errors and iatrogenic complications inherent to spine surgery.

## Introduction

The proclivity for error in routine tasks has been publicly and extensively discussed, and the efficacy of checklists has been particularly validated for aviation [1-3]. In their seminal work, Zuckerman et al. noted that “morbidity due to avoidable medical errors is a crippling reality intrinsic to healthcare; in particular, iatrogenic surgical errors lead to significant morbidity, decreased quality of life, and attendant costs”, and moreover, that the field of neurosurgery has minimally addressed the utilization of checklists [4]. The World Health Organization (WHO) issued the Surgical Safety Checklist and Implementation Manual in 2008 [5]. Since this time, checklists have grown to become an integral part of surgical practice.

Preoperative checklists, usually administered by the nursing staff, exist within every surgical unit to ensure congruency of a patient, planned surgery, correct side and level, and appropriate level of anesthesia, but do not address the most common intraoperative omissions relating to the placement of instrumentation, correction of deformity and wound closure - errors that are common, correctable intraoperatively, and which are usually not reflected in the morbidity reports. These technical omissions, in part, result from the complexity of spine surgery, and may directly impact the surgical outcome. The need for a more comprehensive surgery checklist has been noted [4]. The authors herewith present the Neurosurgical Intraoperative Checklist for surgery of the spine and craniocervical junction, which they have developed during the past decade. 

## Technical report

**Figure 1:**
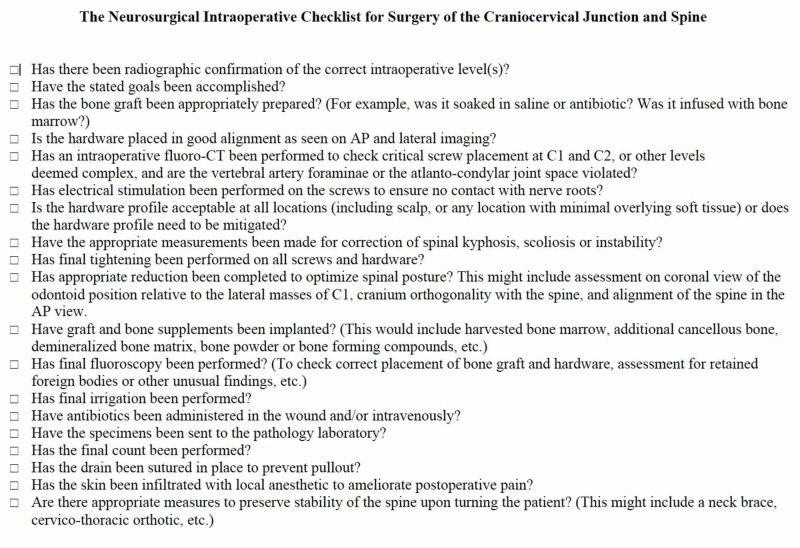
The Neurosurgical Intraoperative Checklist for surgery of the craniocervical junction and spine Created by Henderson Sr FC, Rosenbaum R, Narayanan M, and Mackall JL 2020 AP - anteroposterior

The authors employ a checklist which can be called out by the circulating nurse or by any trusted member of the team. The intraoperative checklist is administered before wound closure begins. The checklist comprises the following questions (see also Figure 1 in Appendix):

- Has there been radiographic confirmation of the correct intraoperative level(s)?

- Have the stated goals been accomplished?

- Has the bone graft been appropriately prepared? (For example, was it soaked in saline or antibiotic? Was it infused with bone marrow?)

- Is the hardware placed in good alignment as seen on AP and lateral imaging?

- Has an intraoperative fluoro-CT been performed to check critical screw placement at C1 and C2, or other levels deemed complex, and are the vertebral artery foraminae or the atlanto-condylar joint space violated?

- Has electrical stimulation been performed on the screws to ensure no contact with nerve roots?

- Is the hardware profile acceptable at all locations (including scalp, or any location with minimal overlying soft tissue) or does the hardware profile need to be mitigated?

- Have the appropriate measurements been made for correction of spinal kyphosis - scoliosis or instability?

- Has final tightening been performed on all screws and hardware?

- Has appropriate reduction been completed to optimize spinal posture? This might include assessment on coronal view of the odontoid position relative to the lateral masses of C1, cranium orthogonality with the spine, and alignment of the spine in the anteroposterior (AP) view. 

- Have graft and bone supplements been implanted? (This would include harvested bone marrow, additional cancellous bone, demineralized bone matrix, bone powder or bone forming compounds, etc.)

- Has final fluoroscopy been performed? (To check correct placement of bone graft and hardware, assessment for retained foreign bodies or other unusual findings, etc.)

- Has final irrigation been performed?

- Have antibiotics been administered in the wound and/or intravenously?

- Have the specimens been sent to the pathology laboratory?

- Has the final count been performed?

- Has the drain been sutured in place to prevent pullout?

- Has the skin been infiltrated with local anesthetic to ameliorate postoperative pain?

- Are there appropriate measures to preserve stability of the spine upon turning the patient? (This might include a neck brace, cervico-thoracic orthotic, etc.)

Over the previous six years, approximately 1200 surgeries of the craniocervical junction and spine were performed by the authors (Fraser Henderson and Robert Rosenbaum) at two institutions. There was a 98% compliance in the administration of the checklist; the checklist was inadvertently not administered during occasional emergency spine surgeries. The use of the checklist resulted in the avoidance of the frequent, mundane errors that occur with frequency in complicated surgeries of the spine and craniocervical junction. 

The checklist prompted attention to positioning and both the coronal and sagittal alignment of hardware. Prior to locking down the hardware, we repositioned the Mayfield head holder to optimize craniocervical parameters as previously described and to lessen or eliminate kyphosis, tilt, or rotation [6, 7]. Careful attention to the fluoro-CT images minimized the likelihood of screw misplacement, particularly at C1, C2, C7, and T1. The checklist reminded the team to minimize hardware and bone graft profile to avoid future site pain or wound erosion. The importance of final-tightening and double-checking all hardware is self-evident. Final fluoroscopy was always performed to ensure there were no retained foreign bodies and as a final assurance of sagittal alignment. Mundane issues, such as drain pullout (through lack of a drain stitch), were avoided through the use of the checklist. 

## Discussion

Spine surgery complications remain frequent. For instance, wrong level exposure was reported in 15% of cases in a prospective study of 100 discectomies, whereas wrong side discectomies occurred in 6.8 for every 10,000 procedures performed [8, 9]. Hitherto, neurosurgeons have not vigorously embraced the utilization of checklists [5]. The authors present The Neurosurgical Intraoperative Checklist for use in surgery of the craniocervical junction and spine.

The most technically difficult portions of surgery may understandably draw surgeons’ paramount focus, while the more mundane, repetitive, or simple parts of the procedure often draw less attention. This phenomenon may be more apparent in training programs where the authors believe there could be a higher incidence of small intraoperative omissions, such as failure to double-check final tightening of instrumentation or failure to check all x-rays prior to closure, which not uncommonly result in a return to surgery at a later date. The authors propose that utilization of this intraoperative checklist will minimize technical omissions that would otherwise result in suboptimal outcomes following surgery of the spine and craniocervical junction. 

It is important for the checklist to be administered before closure begins. It is clearly necessary for the surgeons and all operating room staff to halt and pay attention while the checklist is read. This policy we have adopted is concordant with advanced flight safety systems, such as Crew Resource Management, which emphasizes the many parts played by all crew members in air flight safety [10].

Checklists have proven efficacy. Adoption of the WHO surgical safety check-off list, employed in a prospective study of 7,688 patients, accompanied a decrease in complication rate from 11% to 7%, and the death rate from 1.5% to 0.8% [11]. An 88% decrease in surgical errors was demonstrated by DaSilva-Freitas et al. after the implementation of the checklist [12, 13]. Human error is inevitable. While some degree of complication may be inherent to spine surgery, errors must be avoided and corrected whenever possible [14].

## Conclusions

Complex surgery of the spine or craniocervical junction is fraught with great potential for iatrogenic complication, or simply error by omission. The Neurosurgical Intraoperative Checklist is proposed as a practical, rapid and comprehensive means to prevent the common, avoidable errors of spine surgery.

## References

[1] Gawande A (2010). The checklist manifesto.

[2] Gawande AA, Thomas EJ, Zinner MJ, Brennan TA (1999). The incidence and nature of surgical adverse events in Colorado and Utah in 1992. Surgery.

[3] Degani A, Wiener EL (1993). Cockpit checklists: concepts, design, and use. Hum Factors.

[4] Zuckerman SL, Green CS, Carr KR, Dewan MC, Morone PJ, Mocco J (2012). Neurosurgical checklists: a review. Neurosurg Focus.

[5] Organization WH: WHO Surgical Safety Checklist. WHO, Geneva Geneva, Switzerland Switzerland (2009). WHO Surgical Safety Checklist. https://www.who.int/patientsafety/safesurgery/checklist/en/.

[6] Henderson F, Rosenbaum R, Narayanan M, Mackall J, Koby M (2020). Optimizing alignment parameters during craniocervical stabilization and fusion: a technical note. Cureus.

[7] Henderson Sr FC, Francomano CA, Koby M, Tuchman K, Adcock J, Patel S (2019). Cervical medullary syndrome secondary to craniocervical instability and ventral brainstem compression in hereditary hypermobility connective tissue disorders: 5-year follow-up after craniocervical reduction, fusion, and stabilization. Neurosurg Rev.

[8] Ammerman JM, Ammerman MD, Dambrosia J, Ammerman BJ (2006). A prospective evaluation of the role for intraoperative x-ray in lumbar discectomy. Predictors of incorrect level exposure. Surg Neurol.

[9] Devine J, Chutkan N, Norvell DC, Dettori JR (2010). Avoiding wrong site surgery: a systematic review. Spine.

[10] Helmreich RL, Merritt AC, Wilhelm JA (1999). The evolution of Crew Resource Management training in commercial aviation. Int J Aviat Psychol.

[11] Haynes AB, Weiser TG, Berry WR (2009). A surgical safety checklist to reduce morbidity and mortality in a global population. N Engl J Med.

[12] Da Silva-Freitas R, Martin-Laez R, Madrazo-Leal CB, Villena-Martin M, Valduvieco-Juaristi I, Martinez-Agueros JA, Vazquez Barquero A (2012). Establishment of a modified surgical safety checklist for the neurosurgical patient: Initial experience in 400 cases. Neurocirugia.

[13] de Vries EN, Hollmann MW, Smorenburg SM, Gouma DJ, Boermeester MA (2009). Development and validation of the SURgical PAtient Safety System (SURPASS) checklist. Qual Saf Health Care.

[14] Reason J (2000). Human error: models and management. BMJ.

